# Clinically Significant Prostate Cancer Prediction Using Multimodal Deep Learning with Prostate-Specific Antigen Restriction

**DOI:** 10.3390/curroncol31110530

**Published:** 2024-11-15

**Authors:** Hayato Takeda, Jun Akatsuka, Tomonari Kiriyama, Yuka Toyama, Yasushi Numata, Hiromu Morikawa, Kotaro Tsutsumi, Mami Takadate, Hiroya Hasegawa, Hikaru Mikami, Kotaro Obayashi, Yuki Endo, Takayuki Takahashi, Manabu Fukumoto, Ryuji Ohashi, Akira Shimizu, Go Kimura, Yukihiro Kondo, Yoichiro Yamamoto

**Affiliations:** 1 Department of Urology, Nippon Medical School Hospital, Tokyo 113-8603, Japan; s8053@nms.ac.jp (H.T.); s00-001@nms.ac.jp (J.A.); s4036@nms.ac.jp (Y.T.); m-taniuchi@nms.ac.jp (M.T.); h-hasegawa@nms.ac.jp (H.H.); h-mikami86@nms.ac.jp (H.M.); kotaro-o@nms.ac.jp (K.O.); y-endo1@nms.ac.jp (Y.E.); gokimura@nms.ac.jp (G.K.); kondoy@nms.ac.jp (Y.K.); 2 Pathology Informatics Team, RIKEN Center for Advanced Intelligence Project, Tokyo 103-0027, Japan; yasushi.numata@riken.jp (Y.N.); hiromu.morikawa@riken.jp (H.M.); ktsutsum@hs.uci.edu (K.T.); takayuki.takahashi@a.riken.jp (T.T.); manabu.fukumoto.a8@tohoku.ac.jp (M.F.); 3 Department of Radiology, Nippon Medical School Hospital, Tokyo 113-8603, Japan; s7026@nms.ac.jp; 4 Mathematical Intelligence for Medicine, Graduate School of Medicine, Tohoku University, Miyagi 980-8575, Japan; 5 Statistical Genetics Team, RIKEN Center for Advanced Intelligence Project, Tokyo 103-0027, Japan; 6 Department of Integrated Diagnostic Pathology, Nippon Medical School, Tokyo 113-8603, Japan; r-ohashi@nms.ac.jp; 7 Department of Analytic Human Pathology, Nippon Medical School, Tokyo 113-8603, Japan; ashimizu@nms.ac.jp

**Keywords:** deep learning, prostate cancer, clinically significant prostate cancer, multimodal data, PSA

## Abstract

Prostate cancer (PCa) is a clinically heterogeneous disease. Predicting clinically significant PCa with low–intermediate prostate-specific antigen (PSA), which often includes aggressive cancers, is imperative. This study evaluated the predictive accuracy of deep learning analysis using multimodal medical data focused on clinically significant PCa in patients with PSA ≤ 20 ng/mL. Our cohort study included 178 consecutive patients who underwent ultrasound-guided prostate biopsy. Deep learning analyses were applied to predict clinically significant PCa. We generated receiver operating characteristic curves and calculated the corresponding area under the curve (AUC) to assess the prediction. The AUC of the integrated medical data using our multimodal deep learning approach was 0.878 (95% confidence interval [CI]: 0.772–0.984) in all patients without PSA restriction. Despite the reduced predictive ability of PSA when restricted to PSA ≤ 20 ng/mL (*n* = 122), the AUC was 0.862 (95% CI: 0.723–1.000), complemented by imaging data. In addition, we assessed clinical presentations and images belonging to representative false-negative and false-positive cases. Our multimodal deep learning approach assists physicians in determining treatment strategies by predicting clinically significant PCa in patients with PSA ≤ 20 ng/mL before biopsy, contributing to personalized medical workflows for PCa management.

## 1. Introduction

Prostate cancer (PCa) is a clinically heterogeneous disease, which is one of the most commonly diagnosed cancers in elderly men and the sixth leading cause of cancer-related death in Japan [1]. Prostate-specific antigen (PSA) is widely used in clinical practice, leading to a reduction in the risk of cancer spreading and cancer-related deaths [2,3,4]. However, PCa with low–intermediate PSA level also often includes aggressive cancers, such as clinically significant cancer, which can be life-threatening if not addressed [2,3,5]. Clinically significant cancer can progress when appropriate treatment is not performed at the appropriate time.

Artificial intelligence (AI) is gaining considerable attention owing to its excellent performance in medical image classification [6,7,8,9]. In the field of PCa, this technology has achieved notable effects that would be impossible to achieve using conventional approaches. Clinically significant PCa was detected by applying an explainable AI model to prostate magnetic resonance imaging (MRI) [10], showing improved confidence and reading time for non-experts by offering visual and textual explanations using established imaging features. Furthermore, we developed a method to acquire new explainable features from annotation-free histopathological prostate images, which can improve cancer recurrence predictions [11]. Urologists are eager to enhance PCa management in patients with low–intermediate PSA by applying AI technology to predict clinically significant PCa accurately. Our study aimed to predict clinically significant PCa in patients with PSA ≤ 20 ng/mL by employing our deep learning approach on multimodal medical data routinely used in clinical practice without prostate biopsy, which can be used to optimize the overall management of PCa.

## 2. Materials and Methods

### 2.1. Study Design

**Figure 1:**
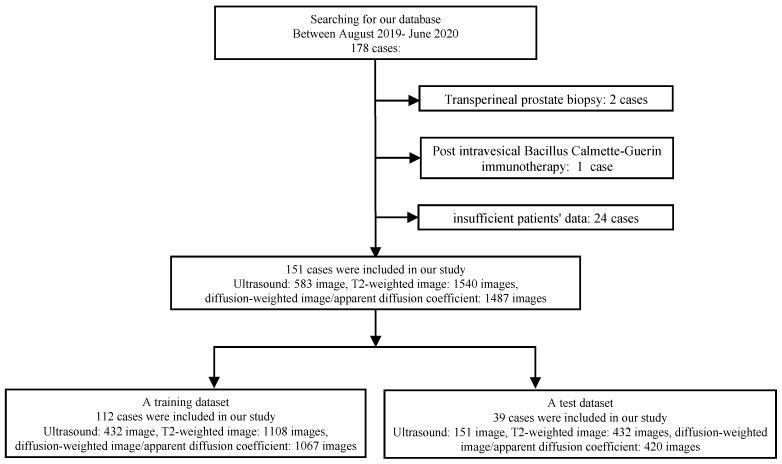
Flowchart of the patient selection procedure.

We enrolled 178 consecutive patients between August 2019 and June 2020. Patients underwent ultrasound-guided prostate biopsy at Nippon Medical School Hospital (NMSH) in Tokyo, Japan. Figure 1 shows the profile used in this study. Cases with transperineal biopsy of the prostate (two cases), history of post-intravesical Bacillus Calmette–Guérin therapy (one case), and others (insufficient saved image and data: 24 cases) were excluded. In our institution, transrectal prostate biopsy was performed in most cases. In this study, we excluded two cases of transperineal biopsy. We evaluated 151 cases, 583 ultrasound images obtained via the transrectal approach, 1540 T2-weighted images (T2WI), and 1487 diffusion-weighted images (DWI)/apparent diffusion coefficient (ADC) using deep learning analysis. Clinical data were divided into two subsets: a training dataset comprising cases between August 2019 and February 2020 and a test dataset comprising cases from March 2020 to June 2020. Clinically significant PCa is variably defined [12], and among several indicators we used the International Society of Urological Pathology (ISUP) prostate cancer grading 2–5 in this study. We evaluated the prediction accuracies for clinically significant PCa before prostate biopsy using the following datasets: PSA, ultrasound imaging, MRI (T2WI, DWI, and ADC), and multimodal clinical data, in whole cases with no restrictions of PSA and in cases with PSA levels ≤20 ng/mL. This study was approved by the Institutional Review Boards of the NMSH (reference O-2021-080) and RIKEN (reference Wako 2023-21). The requirement for informed consent was waived due to the retrospective nature of this study and the lack of intervention. The opportunity to refuse to participate in this study was guaranteed in an opt-out manner via the Ethics Committee of the NMSH website.

### 2.2. MRI Images

All patients underwent biparametric MRI before prostate biopsy. Each scan was performed using a mixed MRI scanner with different gradient strengths (1.5 or 3.0 tesla) with a phased array coil. A previous study revealed that the signal-to-noise and contrast-noise ratios of T2WI were similar at 1.5 and 3.0 tesla. All MRI images were saved in Portable Network Graphics (PNG) format. A rectangular region of the prostate was extracted from these images. This rectangular region included proximate tissues, such as the prostatic capsular vessels, pelvic fascia, and rectum. We adjusted these images to 256 × 256 pixels for the deep learning analysis.

### 2.3. Ultrasound Imaging

Prostate ultrasound imaging was performed at four locations (base, middle, middle-apex, and apex). All ultrasound images were saved in Digital Imaging and Communications in Medicine (DICOM) format. All the DICOM ultrasound images were converted to PNG, and a rectangular section of the prostate was isolated from the images. This rectangular area encompasses neighboring structures such as the prostatic capsular vessels, pelvic fascia, and rectum. The images were resized to 256 × 256 pixels. We used an ultrasound system (Aplio i800; Canon Medical Systems, Tokyo, Japan) with a 6 MHz transrectal probe (PVT-770 RT; Canon Medical System).

### 2.4. Pathological Evaluation

Histopathological assessments were performed by two pathologists in accordance with the ISUP grading [13]. Pathologists independently diagnosed all cases and reached a consensus.

### 2.5. Prediction Using Machine Learning Analysis

**Figure 2:**
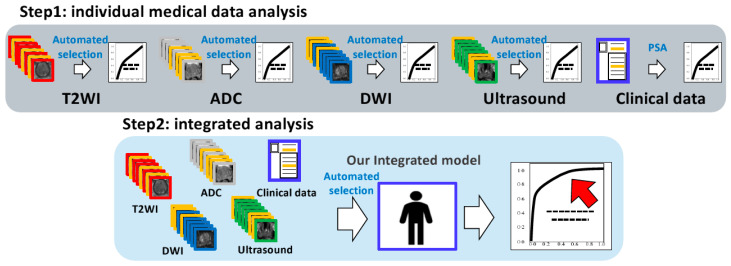
Graphical flowchart of machine learning analysis. Step 1: individual medical data analysis (upper image)—our system selected three images (yellow frame) of each modality based on the method in Section 2.5 (automated selection). Predictive probabilities belonging to each of the three images outputted by neural network are employed as SVM features for prediction. Step 2: integrated analysis (lower image)—similarly, our system selected three images (yellow frame) belonging to each modality based on the method in Section 2.5 (automated selection). A total of 12 predictive probabilities from each modality along with clinical data (PSA) were employed as SVM features for prediction. Abbreviations: SVM: support vector machine, PSA: prostate-specific antigen, T2WI: T2-weighted imaging, ADC: apparent diffusion coefficient, DWI: diffusion-weighted imaging.

We applied a deep convolutional neural network model [14], which was pre-trained on ImageNet. We used an augmentation technique, including a zoom range parameter. We assigned positive or negative labels to these datasets for the analyses (clinically significant PCa or others). Three images were automatically selected in cases with multiple images per patient based on the top three highest probabilities (|P_dl_-0.5|, P_dl_: the predicted probability of deep learning prediction). Our previous study [15] showed that using three suitable images provides the most accurate analysis. We used the predicted probabilities of the deep learning prediction as feature values for multimodal analysis. We summed the features from each modality and employed them as support vector machine (SVM) features for prediction (Figure 2). We constructed a receiver operating characteristic (ROC) curve with the corresponding area under the curve (AUC) to evaluate the predictions. We determined the thresholds using the Youden index. We used the e1071 package (version 1.7.14) of the R software for the SVM. The SVM calculations were performed automatically using software packages.

### 2.6. Statistical Analysis

A Wilcoxon rank-sum test was used to assess the differences in continuous variables. The construction and comparison of ROC curves were performed using the ‘pROC’ package (version 1.18.5) in the R programming language, version 4.4 [16]. All *p*-values reported in this study were two-sided, and statistical significance was determined at *p* < 0.05.

### 2.7. Data Availability

The clinical data analyzed in this study were collected with the cooperation of each patient through medical treatment at NMSH. Protecting their personal information has priority, therefore these data are not publicly available. The data presented in this study are available on request from the corresponding author after approval by the NMSH institutional ethics committee.

## 3. Results

### 3.1. Image and Patient Characteristics

Table 1 shows the 151 patients enrolled in our study, all of whom underwent ultrasound-guided prostate biopsies at the NMSH. We classified cases based on the presence or absence of clinically significant PCa. The median age of all patients was 71 years [interquartile range (IQR): 66–76 years]. The median age of the patients with clinically significant PCa was 72 years (IQR: 68–78), and those with clinically significant PCa were significantly older than those without clinically significant PCa (*p* = 0.003). The median PSA level in all cases was 8.6 ng/mL (IQR: 6.1–14.3). Among clinically significant PCa cases, the median PSA was 9.6 ng/mL (IQR: 7.2–27.1). No significant differences were observed in PSA levels between the clinically significant PCa predictions. Biopsy Gleason scores were distributed as follows: Gleason score 6 (11 cases), 7 (40 cases), 8 (19 cases), 9 (27 cases), and 10 (0 cases). Remarkably, 57.0% of these cases were diagnosed with clinically significant PCa (ISUP 2–5). The results indicated trends consistent with the features observed in cases with PSA ≤ 20 ng/mL (Table 1).

### 3.2. Prediction of Clinically Significant PCa

**Figure 3:**
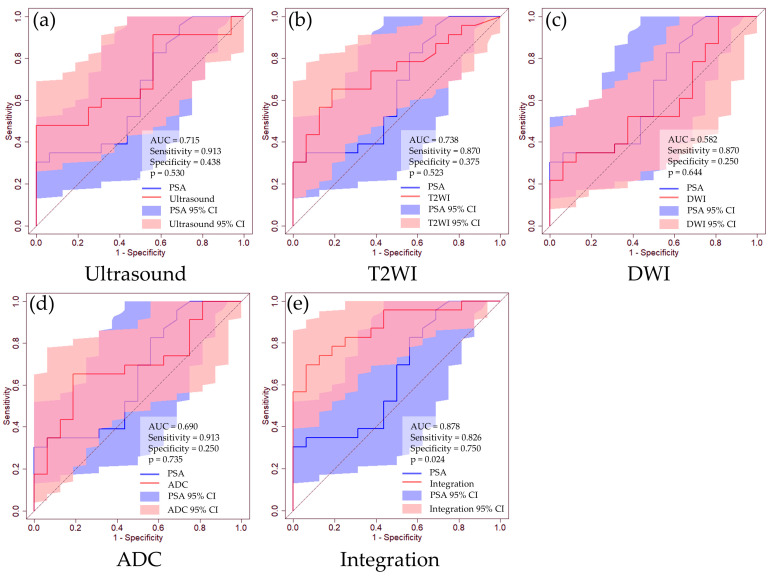
ROC curves of clinically significant PCa prediction using routine clinical data. (**a**) Ultrasound image, (**b**) T2WI, (**c**) DWI, (**d**) ADC, (**e**) integrated medical data. The blue line represents the ROC curve for the PSA level, while the red line corresponds to the ROC curve for each dataset. The blue-shaded region indicates the 95% CI for PSA, and the red-shaded regions represent the 95% CIs for each dataset. We determined the thresholds using the Youden index.

**Figure 4:**
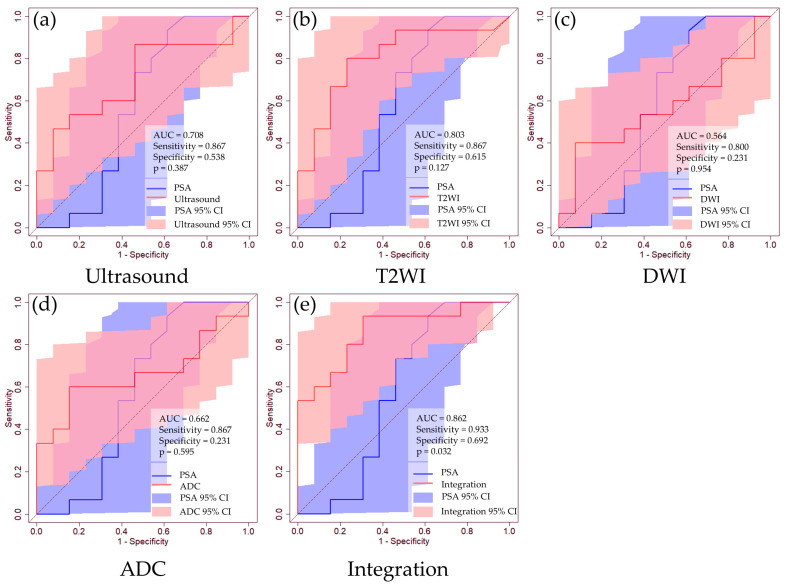
ROC curves for clinically significant PCa prediction in patients with PSA < 20 ng/mL using routine clinical data. (**a**) Ultrasound image, (**b**) T2WI, (**c**) DWI, (**d**) ADC, (**e**) integrated medical data. The blue line represents the ROC curve for the PSA level, whereas the red line corresponds to the ROC curve for each dataset. The blue-shaded region indicates the 95% CI for PSA, and the red-shaded regions represent the 95% CIs for each dataset. We determined the thresholds using the Youden index.

Table 2 shows the prediction accuracies for clinically significant PCa predictions in cases with no PSA restrictions. The AUC values for clinically significant PCa predictions are as follows: PSA, 0.649 [95% CI: 0.467–0.832]; ultrasound imaging, 0.715 (95% CI: 0.551–0.878); T2WI, 0.738 (95% CI: 0.581–0.895); DWI, 0.582 (95% CI: 0.396–0.767); and ADC, 0.690 (95% CI: 0.519–0.861). The integrated analysis demonstrated a remarkable AUC of 0.878 (95% CI: 0.772–0.983). Although individual diagnostic tests did not exhibit statistically significant differences in the AUC compared to PSA, integrated analysis significantly surpassed the results of PSA (*p* = 0.024) (Figure 3). Furthermore, we evaluated the accuracy of clinically significant PCa predictions in cases with PSA levels ≤20 ng/mL. The AUC values for clinically significant PCa predictions are as follows: PSA, 0.574 [95% CI: 0.330–0.819]; ultrasound imaging, 0.708 (95% CI: 0.508–0.908); T2WI, 0.803 (95% CI: 0.629–0.976); DWI, 0.564 (95% CI: 0.341–0.787); and ADC, 0.662 (95% CI: 0.449–0.874). The integrated analysis demonstrated an AUC of 0.862 (95% CI: 0.723–1.000) (Table 2 and Figure 4).

We further assessed clinical presentations and images belonging to representative false-negative and false-positive cases, as described below. False-negative case: a 71-year-old man who was referred to our department after a health check showed a PSA level of 7.0 ng/mL. Pre-biopsy MRI revealed no sign suggestive of malignancy; however, it indicated the presence of benign prostatic hyperplasia. Subsequent biopsy revealed PCa (ISUP 2). False-positive case: a 75-year-old man who was referred to our department after a health check showed a PSA level of 7.5 ng/mL. Pre-biopsy MRI revealed no sign suggestive of malignancy; however, it indicated the presence of benign prostatic hyperplasia. Subsequent biopsy revealed chronic inflammatory changes. Both cases had PSA levels below 10 ng/mL.

## 4. Discussion

This study assessed the predictive accuracy of deep learning analysis by utilizing multimodal medical data to identify clinically significant cancer in patients with a low–intermediate PSA level, specifically below 20 ng/mL, before undergoing prostate biopsy. The predictive performance for clinically significant PCa, measured by the AUC, was 0.862 when integrating medical datasets.

In clinical practice, PCa can have a diverse course, ranging from indolent to aggressive, rapidly progressing, life-threatening tumors. The necessity to accurately diagnose cancer and appropriately treat the disease is essential. Pathological grading is still one of the most prognostic factors for stratifying PCa, and treatment options are proposed according to pathological grading. In 1994, Epstein et al. published the first criteria for defining clinically significant PCa [17]. Clinically significant cancer has a meaningful impact on a patient’s health and requires definitive intervention or treatment. Treatment options for localized PCa include radical prostatectomy, radiation therapy, hormonal therapy, or a combination of these therapies [15,18]. The introduction of robotic surgery systems has expanded the indication of clinically significant PCa. In addition, a combination of external beam radiation therapy, brachytherapy, and hormonal therapy is effective for clinically significant PCa [19,20].

A pressing need to improve diagnostic accuracy to deliver these treatments for clinically significant cancers effectively exists. Efforts to discover new biomarkers for clinically significant PCa have been advancing. The prostate health index (PHI; Beckman Coulter, Brea, CA, USA) score offers a more comprehensive insight into elevated PSA levels and the probability of detecting PCa by biopsy. A meta-analysis of 60 studies involving 14,255 patients observed that the PHI showed a combined sensitivity of 0.874 (95% CI 0.803–0.923) and specificity of 0.569 (95% CI 0.458–0.674) in detecting clinically significant PCa [21]. Prostate cancer antigen 3 (PCA3) encodes a prostate-specific messenger ribonucleic acid that serves as the target for a urine-based diagnostic biomarker for PCa detection. ROC curve analysis revealed that PSA alone resulted in an AUC of 0.63 for PCa detection, whereas a combined PSA and PCA3 score resulted in an AUC of 0.71 [22]. Furthermore, AI technology is anticipated to be pivotal in cancer management. Jin et al. [23] used a T2-weighted imaging-based deep learning method to predict noninvasive PCa detection and Gleason grade. Wang et al. [24] used ADC maps and MRI deep learning to predict the biochemical recurrence of advanced PCa. These datasets in the studies mentioned above are single-modality. In addition, Lombardo R et al. [25] warned about the quality of AI by analyzing the appropriateness of ChatGPT’s response to the European Association of Urology (EAU) 2023 PCa guidelines. A growing trend toward conducting multimodal AI studies has been observed [15]. Zhao et al. [26] and Li et al. [27] used the findings on multiparametric MRI images, and a deep learning approach was conducted to predict significant cancer. However, these previous studies did not focus on patients with low–intermediate PSA. Our multimodal approach for predicting clinically significant PCa in patients with low–intermediate PSA levels, incorporating biparametric MRI alongside ultrasound imaging and clinical data, achieved an AUC of 0.862. Our study focused on patients with PSA ≤ 20 ng/mL; our method may produce robust results regardless of PSA levels.

AI technologies are being developed for practical clinical applications. When integrating AI into healthcare, assessing its suitability for actual medical workflows and carefully judging its appropriateness is crucial [28]. Medical workflows contribute to improving the efficiency and accuracy of medical procedures and play a role in reducing medical errors. In this study, we only utilized medical data that are practical and available in clinical settings to deploy AI technologies without disrupting the current workflow. Our method may advance cancer management through effective analysis of medical big data.

The main limitation of this study was that it was conducted at a single facility with a relatively small sample size (178 patients). However, we analyzed 583 ultrasound images, 1540 T2-weighted images, and 1487 DWI or ADC images. Furthermore, we applied augmentation techniques and transfer learning based on ImageNet32 [29]. In the future, we intend to obtain a validation dataset from external data and perform subsequent analyses. Expanding the dataset could improve the accuracy of the prediction analysis. In addition, we found patterns of benign prostatic hyperplasia among representative images belonging to both false-negative and false-positive cases. Increasing the number of training images may be useful for classification of benign prostatic hyperplasia patterns. Further research is needed to strengthen our findings. Moreover, while this study provides initial insights, using a larger cohort with other significant PCa labels which includes transperineal biopsy cases would enhance the generalizability of our findings given that we used only transrectal prostate biopsy samples in this study.

## 5. Conclusions

Our study illustrates that multimodal deep learning may assist in identifying clinically significant PCa in patients with low–intermediate PSA levels before prostate biopsy. Urologists may enhance personalized workflows for managing PCa by integrating medical data through AI technology.

## Figures and Tables

**Table 1:** Patient characteristics for clinically significant PCa predictions.

Characteristics	No PSA Restriction				PSA < 20 ng/mL			
	Total	Clinically Significant PCa	Others	*p*	Total	Clinically Significant PCa	Others	*p*
Cases, *n*	151	86	65	-	122	71	51	-
Age (years)								
Median (IQR)	71, 66–76	72, 68–78	68, 63–73	0.003	71, 65–75	72, 69–78	68, 63–73	0.009
PSA (ng/mL)								
Median (IQR)	8.6, 6.1–14.3	9.6, 7.2–27.1	7.5, 4.8–11.1	0.195	7.7, 5.6–10.4	7.8, 6.4–9.8	7.1, 4.7–10.7	0.261
Gleason score	6: 11, 7: 40, 8: 19. 9:27, 10: 0				6: 11, 7: 39, 8: 14. 9:7, 10: 0			

PCa: prostate cancer, IQR: interquartile range, PSA: prostate-specific antigen.

**Table 2:** AUCs of the clinically significant PCa prediction.

Variables	No PSA Restriction (*n* = 151)			PSA ≤ 20 ng/mL (*n* = 122)		
	AUC	95% CI	*p*	AUC	95% CI	*p*
PSA	0.649	0.467–0.832	-	0.574	0.330–0.819	-
Ultrasound	0.715	0.551–0.878	0.530	0.708	0.508–0.908	0.387
T2WI	0.738	0.581–0.895	0.523	0.803	0.629–0.976	0.127
DWI	0.582	0.396–0.767	0.644	0.564	0.341–0.787	0.954
ADC	0.690	0.519–0.861	0.735	0.662	0.449–0.874	0.595
Integration	0.878	0.772–0.984	0.024	0.862	0.723–1.000	0.032

AUC: area under the curve, PCa: prostate cancer, CI: confidence interval, PSA: prostate-specific antigen, T2WI: T2-weighted image, DWI: diffusion-weighted imaging, ADC: apparent diffusion coefficient.

## Data Availability

The clinical data analyzed in this study were collected with the cooperation of each patient through medical treatment at NMSH. Protecting their personal information has priority, therefore these data are not publicly available. The data presented in this study are available on request from the corresponding author after approval by the NMSH institutional ethics committee.
